# A comparison of sleep, depressive symptoms, and parental perceptions between U.S. and Taiwan adolescents with self-reported sleep problems

**DOI:** 10.1093/sleepadvances/zpaa004

**Published:** 2020-09-14

**Authors:** Ya-Ting Yang, Katherine A Kaplan, Jamie M Zeitzer

**Affiliations:** 1 Institute of Education, National Cheng Kung University, Tainan City, Taiwan, ROC; 2 Department of Psychiatry and Behavioral Sciences, Stanford University, Stanford, CA; 3 Mental Illness Research Education and Clinical Center, VA Palo Alto Health Care System, Palo Alto, CA

**Keywords:** adolescent, sleep, sleepiness, depression, circadian rhythm, chronotype, parental perceptions

## Abstract

**Study Objectives:**

Inadequate sleep is pervasive among teens worldwide, resulting in daytime sleepiness and, in some cases, depressive symptoms. In addition to their own behavioral choices, parent perceptions may also play a role in adolescent sleep. This study conducted a preliminary evaluation of the antecedents and consequences of sleep factors among adolescents in the United States and Taiwan.

**Methods:**

Participants were adolescents with self-reported sleep concerns from academically similar schools in Taiwan (*n* = 548) and northern California, United States (*n* = 128). Questionnaires on sleep and mood were administered to both the teens and parents.

**Results:**

While Taiwanese students’ self-reported sleep behavior was generally better than U.S. students (*p* < .01), Taiwanese students had higher overall self-reported sleepiness (*p* < .01). Furthermore, Taiwanese parents reported teen sleep durations of 6.53 ± .827 hours per night during the week (with 45% perceiving this as sufficient), while U.S. parents reported teen sleep durations of 7.22 ± .930 hours (with 27% perceiving this as sufficient). Adolescents in both cohorts had high levels of symptoms consistent with depression (Taiwan: 70%, United States: 62%), which was associated with shorter sleep times for both cohorts and evening chronotype in the Taiwanese, but not U.S., adolescents.

**Conclusions:**

Some differences exist between Taiwanese and U.S. adolescents, with generally better sleep and less sleepiness reported among students in the United States, and Taiwanese students’ sleep influenced more strongly by chronotype. Furthermore, Taiwanese parents reported less concern about their child’s insufficient sleep, despite the fact that inadequate sleep is strongly associated with depressive symptoms for both cohorts.

**Statement of Significance**

While inadequate sleep is endemic among teens worldwide, there have been few direct comparisons among teens in different countries. In comparing high academically achieving teens from the United States and Taiwan, we find that both groups sleep less than recommended amounts, which is not surprising. Both groups also have elevated levels of depressive symptoms. Importantly, however, despite reporting being aware of the inadequate sleep and its daytime sequelae (e.g. frequently falling asleep during class), Taiwanese parents were less concerned about sleep insufficiency than their American counterparts. This points to the importance of culture when attempting in evoke the social changes necessary to increase sleep time in teens.

## Introduction

There has been increased attention placed on the causes and consequences of inadequate sleep [1–3]. One population in whom inadequate sleep duration is endemic is that of high school students, who have early rise times dictated by school start times and late bed times that are likely due to reinforcing biological and behavioral factors [4–6]. Biologically, changes associated with adolescent development may contribute to a delay in sleep onset secondary to a shift in chronotype (i.e. circadian-based preference) to a later hour [7]. Behavioral factors include evening caffeine use, personal electronics (e.g. computers, smartphones, and television) use, homework, extracurricular activities and afterschool jobs, and evening light exposure [8]. It is noteworthy that this transition to eveningness is unlikely to be purely cultural as it has been found in several countries beyond the United States, including Italy, Germany, Japan, and Taiwan [9–13]. It is also important to consider that an adolescent’s choice of bed timing and their perception of the relative importance of sleep is shifting from the parents’ responsibility to that of the child and still may be influenced by a wide variety of factors including, to some degree, parental opinions about sleep, parental enforcement or suggestions of bed times, as well as parental sleep patterns themselves [14–16].

This shift to a later chronotype in adolescents is associated with behavioral/emotional problems, suicidality, and substance use disorders [17]. Of particular note, later chronotypes report more severe depressive symptoms in clinical and healthy samples [18, 19] and are more likely to develop depression [20]. The association between late chronotype and depression could be mediated or exacerbated by the association of late chronotype with insufficient sleep, as short sleep duration is also associated with increased depressive symptoms in adolescents [21].

The interplay of sleep, chronotype, and mood also could be modified by the society in which people live. Chronotype, as determined through self-report or monitoring of habitual sleep patterns, may be highly influenced by requirements of work, family, or school. For example, in Taiwan, where one study indicated 21% of teens identified as evening type [22], nearly half of all high school students receive after-school tutoring in so-called “cram school” [23], which has been independently associated with insufficient sleep [24]. Attendance at cram school pushes homework and then bedtime to later hours. The elevated percent of so-called evening types in Taiwanese teens may therefore be secondary to an enforced behavior rather than innate [22].

In the current study, we aimed to examine differences in chronotype, sleep, and mood in a cohort of Asian (Taiwan) and U.S. (California Bay Area) teens who were matched for attendance at high academically achieving schools. Specifically, the intentions of this study are to evaluate whether there are differences between Taiwanese and U.S. teens in their chronotype, depressive symptoms, sleep behaviors, sleepiness, and their parents’ impression of their sleep.

## Methods

### Participants

Data were collected throughout the academic year from students enrolled full-time in public school systems in the United States (2013–2016) and Taiwan (2017). Participants (United States, *n* = 128; Taiwan, *n* = 548) were high school students, 14–18 years of age. Recruitment was accomplished via ongoing partnerships with local schools, pediatricians, the Stanford Sleep Clinic, the Taiwan Society of Sleep Medicine, and posts to online bulletin boards and high school electronic newsletters. Inclusion criteria were enrollment full-time in grades 9–12 and expressing difficulty going to bed earlier and waking up early enough. Exclusion criteria included taking medication specifically for sleep disorders. In both countries, participants were from heterogeneous populations in terms of socioeconomic status and were from high ranking schools (average Scholastic Aptitude Test scores for the U.S. schools were in the 87th national percentile; students in Taiwan achieved at least 90th percentile ranking on the High School Entrance Exam). In the United States, participants were recruited from 26 schools in California; whereas, in Taiwan, participants were recruited from eight schools in northern, central, and southern regions. All procedures were approved by the Institutional Review Boards of Stanford University and National Cheng Kung University. Assent was obtained from the adolescent participant and consent was obtained from at least one parent.

### Measures

#### Morningness Eveningness Scale for Children (MESC)

English and Chinese versions of the 10-question MESC were administered to determine chronotype, the measure of the degree of morning or evening preference [17, 25]. This measure, initially validated in 11–12 year olds, has frequently been used in adolescent samples. A recent review of this instrument across 28 studies showed variable internal consistency (*α* = .63–.88) [26], similar to our sample (United States *α* = .74; Taiwan *α* = .70). Following Collado Mateo *et al.* [27], we used cutoff values at the 25th and 75th percentile of the entire cohort to parse chronotypes, which corresponds to values of 10–22 for evening type, 23–27 for intermediate type, and 28–43 for morning type.

#### Center for Epidemiological Studies Depression Scale for Children (CESDC)

English and Chinese versions of the 20-question CESDC were administered to measure depressive symptoms [28, 29]. CESDC scores range from 0 to 60, with higher scores indicating increased levels of depressive symptoms; a cutoff score of 15 is suggestive of depressive symptoms in children and adolescents. Reliability in our sample was good (United States *α* = .89; Taiwan *α* = .90) [30]. In addition to examining the CESDC as a continuous measure, we also dichotomized the samples into those having, or not, symptoms consistent with depression (CESDC >15) [28].

#### Adolescent Sleep Wake Scale (ASWS)

The English version of the 28-question ASWS was administered to U.S. participants to measure self-reported sleep quality [31]. The scale is well-validated, and yields an overall score along with five subscales: going to bed, falling asleep, maintaining sleep, reinitiating sleep, and returning to wakefulness. The original validation data suggested good reliability for the overall score (*α* = .80–.86) and poor to good reliability for the subscales (*α =* .60–.82). For Taiwanese participants, we created a Chinese version through multiple iterations of forward translation, back-translation, expert committee, and pilot testing that showed a similar internal consistency (U.S. overall score *α* = .86, subscale scores *α* = .74–.81; Taiwan overall *α* = .83, subscale scores *α* = .69–.80).

#### Cleveland Adolescent Sleepiness Questionnaire (CASQ)

The English version of the 16-question CASQ was administered to U.S. participants to measure daytime sleepiness in adolescents [32]. The questionnaire is well-validated, and yields an overall score along with four subscales: sleepiness in school, alertness in school, sleepiness during the evening, and sleepiness during transport. The original validation data suggest good reliability for the overall score (*α* = .89). For Taiwanese participants, a Chinese version of the CASQ was created through multiple iterations of forward translation, back-translation, expert committee, and pilot testing. The CASQ is well-validated and the internal consistency in our sample was good (United States *α* = .88; Taiwan *α* = .86).

#### Child and Adolescent Sleep Checklist for parents (CASC-P)

The English version of the 24-question CASC-P was administered to U.S. participants to measure one of the parent’s impression of their teen’s sleep [33]. The questionnaire assesses the overall parental-impression of sleep disturbance, including bedtime problems, breathing during sleep, parasomnias, and daytime problems. A Chinese version of the CASC-P was created for Taiwanese participants through multiple iterations of forward translation, back-translation, expert committee, and pilot testing. Reliability in our sample was adequate (United States *α* = .68; Taiwan *α* = .72).

### Data analyses

Categorical data were compared with chi-square tests for homogeneity, while continuous data were compared with independent samples *t*-tests. Effect sizes (partial eta-squared or Hedges’ *g* or *W*) were reported for all comparisons. Effect sizes can be categorized as weak (.01 ≤ *η*^2^ < .059; *g* ≈ .2; *W* ≈ .1), moderate (.059 ≤ *η*^2^ < .138; *g* ≈ .5; *W* ≈ .3), or strong (*η*^2^ ≥ .138; *g* ≈ .8; *W* ≈ .5) [34, 35]. Other statistical tests are indicated in the text. All statistical analyses were performed by SPSS 20.0 (IBM, New York). The statistical significance level was set at *p* < .05. Data are presented as mean ± SD.

## Results

### Demographics

Students were drawn from high achieving schools in the United States (26 in the general area of the San Francisco Peninsula) and Taiwan (8 schools in northern, southern, and central regions). The cohorts from the United States and Taiwan were similar in terms of distribution of grade, gender, and family income (Table 1).

**Table 1.** Demographics of U.S. (*n* = 128) and Taiwanese students (*n* = 548)

	United States		Taiwan		
	%	*n*	%	*n*	
Grade*					
9	31.0	39	26.8	147	*χ* ^2^ = 6.80
10	33.3	42	25.2	138	*p* = .08
11	18.3	23	22.5	123	
12	17.5	22	25.6	140	
Gender					
Female	50.8	65	51.6	283	*χ* ^2^ = 0.03
Male	49.2	63	48.4	265	*p* = .86
Family income^†^					
High	67.7	67	60.3	323	*χ* ^2^ = 3.88
Medium	27.3	27	28.6	153	*p* = .14
Low	5.1	5	11.2	60	

*Missing data, *n* = 2 (United States).

^†^Grouped as <$50 000, $50 000–$150 000, >$150 000 annual income in the United States and grouped based on self-reported income and education, employment status, and occupation in Taiwan [45]; missing data, *n* = 29 (United States), *n* = 12 (Taiwan).

### Chronotype

Students in the United States had lower scores on the MESC, indicating a greater evening preference in the U.S. cohort (Table 2). This was borne out in categorical examination of chronotypes as well. Of the students in the United States, 52% were evening type, 14% morning type, and 34% neither type, while of the students in Taiwan, 24% were evening type, 34% morning type, and 42% neither type (*χ*^2^ = 43.5, *p* < .01, *W* = .25). According to parental report (CASC-P), however, U.S. students, went to sleep earlier than Taiwanese students on weekdays (23:17 ± 57 vs. 23:43 ± 50 minutes; *p* < .01, *t*-test; *g* = .53), but not weekends (24:10 ± 69 vs. 24:02 ± 52 minutes; *p* = .20, *t*-test; *g* = .15). U.S. students also awoke later during weekdays (7:05 ± 33 vs. 6:27 ± 30 minutes; *p* < .01, *t*-test; *g* = 1.2) and weekends (10:04 ± 84 vs. 8:57 ± 96 minutes; *p* < .01, *t*-test; *g* = .72).

**Table 2.** Sleep-related questionnaires in U.S. and Taiwanese students

	United States		Taiwan			
	Mean	SD	Mean	SD	*t*	*η* ^2^
MESC	22.65	4.48	25.52	4.38	6.63*	.06
CESDC	19.23	9.71	21.49	10.05	2.31*	.01
ASWS	18.32	2.94	22.05	2.75	13.62*	.22
Going to bed	3.30	1.05	3.60	1.05	2.87*	.01
Falling asleep	3.76	.94	4.87	.79	13.80*	.22
Maintaining sleep	4.44	.84	5.15	.66	10.31*	.14
Reinitiating sleep	4.68	.87	5.23	.75	7.24*	.07
Returning to wake	2.14	.80	3.19	1.09	10.38*	.14
CASQ	40.05	9.39	41.29	9.67	1.31	.00
Sleep in school	8.19	3.70	11.16	4.14	7.44*	.08
Awake in school	15.82	4.00	14.75	3.76	2.87*	.01
Sleep in evening	8.87	2.47	8.27	2.66	2.30*	.01
Sleep during transport	7.17	2.67	7.11	2.81	.21	.00
CASC-P	17.02	5.84	15.13	6.05	3.16*	.02
Bed time problems	6.12	2.10	4.53	2.15	7.49*	.08
Sleep breathing/unstable sleep	2.72	2.22	2.92	2.15	.92	.00
Daytime problems	7.20	3.18	6.60	3.13	1.93	.01
Parasomnia/sleep movement	.97	1.43	1.08	1.42	.77	.00

*p < .05.

### Depressive symptoms

Students in Taiwan had higher scores on the CESDC, indicating a greater amount of depressive symptomatology in the Taiwanese cohort (Table 2). We dichotomized each cohort based on whether scores on the CESDC were consistent with a likelihood of depression. Of the students in the United States, 62% met this criterion for depression and of the students in Taiwan, 70% met this criterion for depression. There was no difference in the rates of meeting depression criteria in the two cohorts (*χ*^2^ = 3.01, *p* = .08, *W* = .07). Chronotype was associated with both degree of depressive symptoms (CESDC score) as well as percentage of individuals meeting criteria for likely depression, with evening chronotype being associated with greater rates of depression and higher average values on the CESDC (Table 3).

**Table 3.** Interaction of chronotype with sleep behavior and depressive symptoms in U.S. and Taiwanese students

		Chronotype			
		Morning	Intermediate	Evening	*η* ^2^
CESDC-raw	United States	17.4 ± 10.2	17.9 ± 7.84	20.6 ± 10.6	0.02*
	Taiwan	18.8 ± 9.82	21.5 ± 8.90	25.3 ± 11.1	
CESDC-criteria	United States	56%	59%	65%	n/a**
	Taiwan	61%	71%	80%	
ASWS	United States	19.7 ± 2.83	19.1 ± 2.86	17.4 ± 2.76	.09**
	Taiwan	23.5 ± 2.43	21.9 ± 2.48	20.2 ± 2.50	
CASQ	United States	36.8 ± 10.0	38.4 ± 7.89	42.1 ± 9.79	.06**
	Taiwan	36.6 ± 8.96	42.1 ± 8.65	46.6 ± 9.29	
CASC-P	United States	19.4 ± 6.84	15.6 ± 5.88	17.3 ± 5.31	.02** ^,†^
	Taiwan	13.9 ± 6.29	14.6 ± 5.59	17.7 ± 5.80	

CESDC-raw, Center for Epidemiological Studies Depression Scale for Children, raw total scores; CESDC-criteria, percentage of individuals with CESDC scores consistent with depression. Continuous data are presented as mean ± SD with *p*-values derived from two-way analysis of variance. Categorical data are presented as percentage with *p*-values derived from *χ*^2^ tests.

*p < .05, **p < .01, ^†^*p* < .05 for interaction.

### Sleep-related behavior

Students in Taiwan had higher scores on the ASWS, indicating better sleep-related behavior in the Taiwanese cohort (Table 2). The ASWS can be divided into five subscales of behavior: going to bed, falling asleep, maintaining sleep, reinitiating sleep, and returning to wakefulness. On each of these subscales, scores in the Taiwanese cohort were greater than the U.S. cohort. On the falling asleep subscale (i.e. how easy it is for individuals to fall asleep), the difference between the cohort from Taiwan and United States was of a large effect size; other subscales were either weak or moderate. We also observed chronotype differences in the ASWS, with worse sleep-related behavior in the evening types in both cohorts (Table 3).

### Sleepiness

Overall self-reported sleepiness scores on the CASQ were similar between the cohorts (Table 2). The CASQ can be divided into four subscales: sleepiness in school, alertness in school, sleepiness in the evening, and sleepiness during transit. Taiwanese students scored higher than U.S. students on sleepiness in school. Taiwanese students also scored lower than U.S. students on alertness in school and sleepiness in the evening; sleepiness during transit was similar. Thus, while the overall CASQ scores were statistically indistinguishable between the cohorts, Taiwanese students were sleepier during the day while U.S. students were sleepier in the evening. We also observed chronotype differences in the CASQ, with greater sleepiness in the evening types in both cohorts (Table 3).

### Parent impression of adolescent sleep

Parents of adolescents in Taiwan had lower scores on the CASC-P than parents of adolescents in the United States, indicating that the parents in Taiwan reported that their adolescent child had fewer problems with sleep (Table 2). On the CASC-P subscale of presleep behaviors, parents from the United States had more concerns regarding their adolescent’s presleep behavior than parents from Taiwan. There were no differences between the cohorts of parental concerns of adolescent behavior during the sleep period, sleep-related behavior during the daytime, or parasomnia and sleep movement. Among the six presleep behaviors captured on the CASC-P (except one concerning leg movement), answers to each of the questions were highly divergent between the cohorts. Parents felt that students always or sometimes used caffeine before bed more in Taiwan (22% vs. 10%, *χ*^2^ = 42.3, *p* < .0001), always or sometimes used electronics before bed more in the United States (94% vs. 79%, *χ*^2^ = 31.0, *p* < .0001), always or sometimes avoided bedtime more in the United States (70% vs. 23%, *χ*^2^ = 126, *p* < .0001), always or sometimes had bedtime anxiety more in the United States (9% vs. 4%, *χ*^2^ = 13.7.3, *p* < .01), and always or sometimes had trouble falling asleep when alone more in the United States (18% vs. 3%, *χ*^2^ = 46.1, *p* < .0001). The frequency that caffeine or electronics was used prior to bed was not associated with parent-reported short (<20 minutes) or long (>20 minutes) sleep latency in either cohort (*p*s > .06, *χ*^2^ test).

We also observed chronotype differences in the CASC-P (Table 3). A chronotype by cohort interaction was present such that the parents of teens from Taiwan who were of morning or intermediate chronotype had lower concerns about their teen’s sleep, as compared with the parents of teens who were evening type (from either cohort, *p*s < .02, Tukey post hoc) or the parents of teens who were morning type (from the U.S. cohort, *p*s < .02, Tukey post hoc).

**Figure 1.**
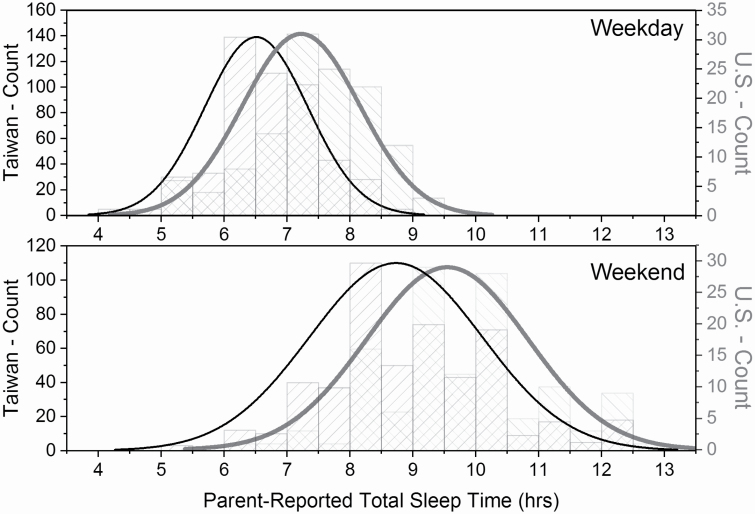
Parent-reported total sleep time (hours) of adolescents on weekdays (upper) and weekends (lower). The histogram for the distribution of responses from the Taiwan cohort are shown with columns (diagonal lines, lower left to upper right) and normal curves in black. The histogram for the distribution of responses from the U.S. cohort are shown with columns (diagonal lines, upper left to lower right), and normal curves in dark gray. Crosshatched columns are indicative of overlap between the two populations.

The CASC-P also reports on parent impression of the adolescent’s sleep quantity (Figure 1). Parents in Taiwan reported that their children slept 6.53 ± .827 hours per night during the week and 8.75 ± 1.37 hours per night on the weekends. Parents in the United States reported that their children slept 7.22 ± .930 hours per night during the week and 9.55 ± 1.25 hours per night on the weekends. Both parent-reported weekday (*p* < .001) and weekend (*p* < .001) sleep duration were shorter in the adolescents in Taiwan (Figure 1). Only 7% of the adolescents in Taiwan and 30% of the adolescents in the United States were reported to have the National Sleep Foundation recommended 8 or more hours in bed. Sleep onset latency in the U.S. cohort was also greater, as 27% of parents reported their child taking more than 40 minutes to fall asleep, as compared with 5% of Taiwanese parents (*χ*^2^ = 99.4, *p* < .0001).

There was divergence on parental concern for sleep as well. In the U.S. cohort, 27% of parents reported feeling that their teen got enough or almost enough sleep, while in the Taiwan cohort, 45% of parents reported feeling that their teen got enough or almost enough sleep (*χ*^2^ = 13.9, *p* < .001). In the parents who reported feeling that their teen got enough or almost enough sleep, the parents reported that the teen slept for 7.95 ± .668 hours in the United States, but only 6.85 ± .775 hours in Taiwan, a difference of 66 minutes (*p* < .001).

### Sleep, sleepiness, and depressive symptoms

In both cohorts, there was a moderate correlation between sleepiness (CASQ total score) and depression (CESDC total score) such that increased sleepiness was associated with increased depressive symptoms (Table 4). In both cohorts, there was also a weak correlation between parent-reported weekday sleep length and depression such that shorter sleep was associated with increased depressive symptoms (Table 4). We did not observe such an association between depressive symptoms and parent-reported weekend sleep length (Table 4).

**Table 4.** Correlation among sleep, sleepiness, and depressive symptoms in U.S. and Taiwanese students

	TST_week_	TST_weekend_	CASQ
Taiwan CESDC	−.15**	.07	.35**
U.S. CESDC	−.20*	−.097	.41**

Pearson *r*-values. TST_week_, parent estimated teen usual total sleep time on a week night; TST_weekend_, parent estimated teen usual total sleep time on a weekend night.

*p < .05, **p < .01.

## Discussion

Our data indicate that in both the United States and Taiwan, adolescents from high achieving high schools who self-identify as individuals with problematic sleep do indeed exhibit insufficient sleep, with only, according to parent report, 7% of the adolescents in Taiwan and 30% of the adolescents in the United States achieving the National Sleep Foundation-set minimum goal of at least 8 hours in bed [36]. The greater proportion of Taiwanese teens having inadequate time in bed is likely secondary to a weekday bedtime that is approximately 30 minutes later and a weekday wake time that is approximately 30 minutes earlier as compared with U.S. teens. Consistent with having inadequate sleep, the Taiwanese adolescents, as compared with those from the United States, were more readily able to go to sleep, from both the adolescents’ and parents’ perspectives, and were sleepier during the daytime. This difference in sleepiness was especially pronounced in adolescents who were of intermediate or evening chronotype.

Nearly half of the parents from Taiwan felt that their teens’ sleep was adequate, despite reporting their teens were obtaining fewer than 7 hours of sleep per night; conversely, only a quarter of the parents from the United States felt that their teens’ sleep was adequate, despite reporting their teens averaged around 8 hours per night. This suggests a difference in expectations for normal sleep durations in teens between parents in the Taiwanese and U.S. cohorts. Such a difference was observed previously in Asian-Australian and Caucasian-Australian parents of younger children, where the former group indicated that children needed less sleep than the latter group [37]. The current study was not designed to specifically answer the question of cultural expectations of sleep in teens, but future studies should address whether such differences exist and whether changes in parental expectations could help drive changes in sleep in teens in Taiwan.

Despite their earlier bedtimes, U.S. adolescents self-reported greater eveningness than the Taiwanese adolescents in this study. This result is different than that of our previous study [22] in which the rates of eveningness were greater than those typically reported for U.S. teens [38]. A possible reason for this difference is that the participants from Taiwan in this study were from high ranking schools (at least 90th percentile ranking on the High School Entrance Exam), while in the previous study, the participants included all levels of academic achievement. Morning-type students, who have been shown to have better learning outcomes in Taiwan [39], may be enriched at the high ranking schools due to the use of entrance exams. Future research could examine whether helping Taiwanese adolescents adjust or regulate their circadian rhythms to adapt to an earlier hour, through either educational interventions or with the assistance of technology such as smart lighting systems, would improve educational outcomes.

U.S. adolescents also reported greater evening sleepiness. Despite this increase in evening sleepiness, they also had an increased difficulty initiating sleep, perhaps secondary to either attempting to go to sleep at an inappropriate circadian phase or presleep behavior that could be activating (e.g. use of certain electronic media). The comparatively reduced sleepiness in Taiwanese teens may have been secondary to their greater use of caffeine before bedtime, which could have masked their self-reported sleepiness. It is of further interest to note that the increased use of caffeine before bedtime in the Taiwanese adolescents was not correlated with increased difficulties in initiating sleep, a finding that we have previously observed in U.S. college students [40], and which may be secondary to their heavy sleep burden. This heavier sleep burden may have also contributed to the parental report of the Taiwanese teens having a relatively easier time initiating sleep, though whether this was culturally influenced was not explored in this study.

While this study recruited adolescents who were concerned with their sleep, we incidentally found very high rates of depressive symptomatology, with approximately two-thirds of both the U.S. and Taiwanese teens reporting elevated symptoms that are consistent with depression. A later chronotype was associated with increased depressive symptoms in the Taiwanese teens, but not in the U.S. teens. Depressive symptoms in both cohorts were associated with self-reported sleepiness and, though less so, time allotted for sleep during the week, but not the weekend. There is a substantial literature that shows an association between disrupted or insufficient sleep and depression [41]. Insufficient sleep is also often found in teens who exhibit suicidality or those who successfully complete suicide [42]. Given the high degree of comorbidity, it would be important to examine depression more directly in teens with concerns about their sleep, especially as treatment of sleep disorders or insufficient sleep may be a more palatable therapy than treatment of depression for some teens. The relationship between increasing sleepiness, decreasing parent-reported sleep duration, and increasing depressive symptomatology suggests that sleep may be a modifiable treatment target on the pathway to addressing the impact of adolescent depression.

The study sample in both Taiwan and the United States was selected specifically because of their concern about their sleep. We do not know whether the rates of insufficient sleep that we observe would be found in teens who do not express concern for their sleep, though we hypothesize they would be lower. Similarly, the rates and association we observe between sleep and depression cannot be extrapolated to a population level. Another shortcoming of the study is that we did not have either objective determination of sleep nor did we have direct self-reported determination of sleep times in the teens. For our analyses on sleep times, we relied on parent report, which may be biased or inaccurate depending on a variety of factors. In one study of Australian parents and their adolescent children, Short *et al.* actually found that parental report tended to idealize sleep which was actually far more curtailed than parents reported [43]. If this were the case in our cohorts, then the degree of sleep loss would be even greater than that which we observed. Parental perception of their adolescent child’s sleep patterns may, however, be culturally dependent. It will be crucial for future studies to examine how a biased estimation of a child’s sleep pattern might influence the opinion and behavior of the parent.

We do, however, show that in cohorts of teens from both Taiwan and the United States who are from high achieving schools and are concerned about their sleep, their concerns are an accurate reflection of curtailed total sleep time as well as being associated with elevated depressive symptomatology. Starting with the assumption that most of the parents of the teens who we studied were interested in the academic outcomes of their children, especially as most were from high achieving schools, it will be critical for future research to identify the trade-off between increased time spent on academics (both in school and after school) and time allocated for sleep. As insufficient sleep can lead to poor academic performance in teens [44] through both direct (decreased memory improvements in sleep) and indirect (sleepiness during the daytime reducing attention and memory acquisition) mechanisms, a greater understanding of the balance between sleep and academics may help to inform parents as well as public policy on the importance of sleep for academics, as well as emotional and physical well-being.
